# Short-term acceptability of female condom use among low-fee female sex workers in China: a follow-up study

**DOI:** 10.1186/s12905-019-0773-7

**Published:** 2019-06-14

**Authors:** Chu Zhou, Evelyn Hsieh, Keming Rou, Jonas Tillman, Wei Dong, Xian-xiang Feng, Yan-zhen Yang, Yu-jun Yang, Xian-guo Sun, Hai-jian Zang, Ying-zhen Wu, Zunyou Wu

**Affiliations:** 1 Division of Prevention and Intervention, National Center for AIDS/STD Control and Prevention, Chinese Center for Disease Control and Prevention, 155 Changbai Road, Changping District, Beijing, 102206 China; 2 Department of Internal Medicine, Yale School of Medicine, New Haven, Connecticut USA; 3 Division of HIV Prevention, Liuzhou Center for Disease Control and Prevention, Liuzhou, Guangxi Zhuang Autonomous Region China; 4 Division of HIV Prevention, Zhangjiajie Center for Disease Control and Prevention, Zhangjiajie, Hunan Province China; 5 Division of HIV Prevention, Pingnan Center for Disease Control and Prevention, Pingnan, Guangxi Zhuang Autonomous Region China

**Keywords:** HIV, Female sex workers, Female condoms, Follow-up study, China

## Abstract

**Background:**

Low-fee female sex workers (FSW) lack power to effectively negotiate male condom use with clients. Female condoms (FCs) may provide an alternative strategy. This study was conducted to assess the acceptability of FC use among low-fee FSWs, and to identify appropriate candidates for future FC promotion.

**Methods:**

A one-month follow-up study was conducted. At entry into the study, eligible participants completed a baseline questionnaire and were given 10 FCs. At the one-month follow up encounter, the number of used FC packages were counted and each participant completed a follow-up questionnaire. Logistic regression was used to identify variables associated with more frequent use of FCs (> 2 times).

**Results:**

A total of 312 low-fee FSWs were enrolled at baseline and all participants completed the follow-up evaluation. Among them, 123 (39.4%) participants had used more than two FCs. Participants who were illiterate or had completed at most primary school education (OR: 2.4, 95% CI: 1.4–7.2), charged ≤30 RMB per client (≤30 vs. 51–80 RMB, OR: 3.8, 95% CI: 1.9–7.6), or had consistently used condoms with regular clients in the past month (OR: 2.4, 95%CI: 1.4–4.2) were more likely to use FCs.

**Conclusion:**

Low-fee FSWs charging ≤30 RMB per client, and those who are less educated may be appropriate initial candidates for FC promotion in China. Strategies to consider include teaching FSWs tactics for negotiation of FC use that can initially be applied with regular clients, and providing education to maximize ease-of use, and minimize discomfort with FC usage.

**Electronic supplementary material:**

The online version of this article (10.1186/s12905-019-0773-7) contains supplementary material, which is available to authorized users.

## Background

Condoms have played a key role in tackling the human immunodeficiency virus (HIV) epidemic globally. To increase access to, and promote consistent and correct use of male and female condoms (FCs) among high-risk populations, condoms have historically comprised a key part of strategies for the prevention of STI and HIV transmission [1]. FCs, which can be initiated independently by women, have been shown to provide comparable or improved efficacy against transmission of STIs and HIVs in comparison to male condoms [2, 3].

For vulnerable populations, such as female sex workers (FSWs), FCs may provide an alternative and more feasible option for FSWs to protect themselves, especially when clients refuse to use a male condom [4]. Research has suggested that access to FCs can decrease the number of unprotected sex acts and sexual transmitted infections (STIs) among FSWs, often through a combination of increased MC as well as FC usage [5]. In China, low-fee FSWs typically gather in low-tier venues including but not limited to small beauty salons/massage parlors, small guesthouses, self-rented rooms and rural “market day” buildings, charging less than or equals to 80 RMB per commercial sex [6, 7]. High-fee and medium-fee FSWs usually work in karaoke bars, or hotels, charging more than 80 RMB per commercial sex [8]. Compared with high-fee and medium-fee FSWs, low-fee FSW are known to be at higher-risk for acquiring sexually transmitted infections (STIs) and human immunodeficiency virus (HIV) and consequently also pose a high risk for transmitting these infections to their clients and other sexual partners. A study conducted in 6 cities in southern China found that HIV prevalence differed among low, middle and high-fee FSWs (1.37, 0.28, and 0.07%, respectively) [8]. Syphilis prevalence among low-fee FSWs was also highest at 9.7%, compared with 4.2% among middle-fee and 2.2% high-fee FSWs, in another cross-sectional study conducted in 8 sites across China [9]. Finally, a cross-sectional study conducted in 12 Chinese sites targeting low-fee FSWs showed that the prevalence of HIV for the sample was much higher at 4.7%, compared with national wide HIV prevalence (less than 1%, unpublished data, China National HIV/AIDS Sentinel Surveillance Report, 2014) [7].

For low-fee FSWs, a number of specific obstacles have led to low rates of consistent male condom use (less than 60%) [6, 7]. These barriers include: lack of a gatekeeper (i.e. someone who is present at the work venue to ensure the FSW’s safety and to support, promote and negotiate male condom use on behalf of FSWs) at certain low-fee work venues such as self-rented rooms [10]; economic pressure to support family or children, making low-fee FSW more likely to give up male condom use to avoid losing clients [1, 6, 11] and an older clientele with higher prevalence of erectile dysfunction, making male condom usage technically difficult [12].

In theory, FCs should be promoted as an alternative to male condoms in this population. Potential barriers to FC uptake include usage-related barriers—including condom breakage, invagination, misdirection, or slippage—economic barriers or lack of access to FCs. In addition, lack of acceptability among clients may present a barrier for uptake among FSW [13]. To date, 6 prior studies have explored the acceptability of FC usage among FSWs in 11 different cities in China. Overall, approximately 30–60% of participants in these studies used FCs after distribution, and 20% of those users expressed willingness to use FCs continuously [5, 14–18]. However, these prior studies did not compare utilization among low-fee FSW versus medium- and high-fee FSW.

However, these prior studies were limited in that they were not designed to specifically target low-fee FSWs. Because this vulnerable group bears much higher STI and HIV risk, and exhibit the lowest rates of consistent male condom use, we decided to conduct a focused study of FC usage among low-fee FSWs in order to assess the acceptability of FC use, identify the most appropriate candidates within this population for future FC promotion efforts, and provide guidance on promoting FC use in situations where FSW feel they cannot use male condoms.

## Methods

### Study sites, participants and recruitment

Three study sites in southern and central China: Liuzhou (Guangxi Zhuang Autonomous Region), Pingnan (Guangxi Zhuang Autonomous Region) and Zhangjiajie (Hunan Province), were selected based upon the following criteria: HIV prevalence among low-fee FSWs greater than 2% according to the 2012 national or local HIV surveillance system, and presence of local CDC outreach workers experienced in carrying out health promotion activities targeting low-fee FSWs, and therefore familiar with the study population.

The sample size was estimated by pre-study mapping. From March to April, 2013, local outreach workers went to every low-fee venue where low-fee FSWs typically encounter clients, such as small beauty salons/massage parlors, small guesthouses, self-rented rooms and “market day” buildings, and counted how many low-fee FSWs were present at each venue. After the mapping process, it was estimated that 100 low-fee FSWs per study site could be feasibly enrolled in our study. From May to October 2013, local outreach workers directly recruited low-fee FSWs in these venues. Eligible women included individuals aged 18–60 years who self-identified as a FSW, charged less than or equals to 80 Renminbi (RMB; 1RMB = approximately $0.16 US dollars) per client (price per client throughout this manuscript refers to the price charged for a vaginal sex transaction), and were not planning to change their occupation in the next month. The purpose of the study was verbally explained to all eligible women by the trained local outreach worker, and those who chose to participate completed the written informed consent process.

### Study procedures and measures

Three outreach workers were selected from the local CDC for each city, and all of them were trained sequentially in each city, in a consistent manner by the researchers. The content of the training included introduction to study design, methodology for completing the study questionnaire and correct FC use. At the time of enrollment for each participant, the local outreach worker administered a face-to-face structured questionnaire focused on socio-demographic and work-related characteristics (Additional file 1), including: age (month and year of birth); marital status (single, married. Divorced, widowed, cohabiting), education level (illiterate, primary school, secondary school, high school, college and higher), household registration (local, non-local); length of time engaged in commercial sex worker (years); price charged per sexual act (RMB); and work venues (small beauty salons, massage rooms, guest houses, self-rented rooms, “market day” buildings).

Upon completing the questionnaire, each participant received a practical and visually aided tutorial lasting approximately 10 min on the handling and use of FCs. FSWs were also provided with a pictorial leaflet illustrating how to properly insert and discard FCs, and how to troubleshoot common problems, including improper insertion or penile misrouting. The local outreach worker subsequently distributed a plastic bag with 10 FCs to each participant. Every participant was informed to keep used FC wrappers in the plastic bag after each FC was used so that they could be counted at the follow-up encounter. The female condom used in this study was originally designed and developed by PATH as the Woman’s Condom, and subsequently licensed to Dahua Medical Apparatus Company to manufacture and distribute in China as O’lavie™. O’lavie™ employs a rounded insertion capsule which dissolves once it is put in place, instead of a fixed inner ring, which facilitates insertion. A comparative study of three FCs reported that significantly more women prefer using the Woman’s Condom (PATH) compared with FC2 and V-Amour [19].

Each participant had a second study encounter 30 days after enrollment. Two to 3 days prior to this follow-up visit, the local outreach workers contacted the gatekeepers or study participant via telephone to schedule an appointment time; then on the day of the encounter, the local outreach workers went to the participant’s work venue and conducted the follow-up visit in person. During the follow-up visit, the outreach workers first counted the used FC packages and then administered a structured questionnaire (Additional file 1), which addressed patterns of FC use with different sexual partners (new/regular clients), discomforts encountered with FC use (multiple choice:all choices displayed in Table 3), willingness to use FCs in the future, and reasons for any unwillingness to use FCs in the future (four choices: “[The FC] is complicated and not convenient to use”, “I prefer to use male condoms”, “[FCs] feel uncomfortable inside the vagina”, “My clients refused to use [FCs]”, more than one choice allowed).

### Statistical analysis

Data were checked for accuracy through double data entry in EpiData (version 3.02, The EpiData Association, Odense, Denmark) and analyzed using SPSS (version 21.0, IBM SPSS Statistics, IBM Corporation, New York, U.S.A).

Acceptability of FC use was defined by two indicators: 1) the overall proportion of participants who used at least 1 FC and the total number of FCs used by each FSW, and 2) the proportion of participants who used FCs that reported willingness to use FCs in the future. Descriptive statistics were used to report measures of central tendency and frequencies, including: age (≤ 35 yrs. versus > 35 yrs., participants who were older than 35 were categorized as older FSWs, in accordance with previous studies [20]; marital status (single/widowed/divorced versus married/cohabiting); education (illiterate/primary school versus secondary school and higher); household registration or *hukou* in Chinese (local versus non-local, non-local indicating migration from other regions of China); length of time engaged in commercial sex work (≤ 2 yrs. versus > 2 yrs., more than 2 years as an indicator of ‘long-term FSW’); price charged (≤30 RMB, 31–50 RMB, 51–80 RMB); venue (small beauty salons/small massage rooms/small guest houses versus self-rented room/“market day” buildings, the first category includes venues with a gatekeeper).

Using frequency of FC use in the past month (0–2 times versus > 2 times) as the categorical outcome variable of interest, we performed univariate logistic regression to identify variables that were significantly associated with more frequent FC use. Variables with a significance level of *p < 0.1* were kept as potential candidates for a multivariable logistic regression model. After assessing all significant independent variables for multicollinearity, we removed the variable city (categorical describing which city the individuals were sampled from) from the model due to high correlation with price charged and venue. The final model was identified according to the method “purposeful selection of variables in logistic regression” described by Bursac et al. (with a cut-off *P < 0.05*) [21]. We subsequently stratified the study population by the significantly associated factors identified in our multivariate regression model, and Chi squared testing was used to explore patterns of FC use with different types of sexual partners.

## Results

### Baseline characteristics

A total of 312 low-fee FSWs (comprised of 119, 100 and 93 participants from Liuzhou, Pingnan, and Zhangjiajie, respectively) were enrolled and completed the baseline survey. Baseline characteristics of the participants are shown in Table 1.

**Table 1:** Participants’ Characteristics, and Univariate and Multivariable Analysis of Frequent FC Use in Three Chinese Cities, 2013, *N* = 312

Variables	Total N (%)	0–2 N 1(%)	> 2 N2 (%)	Univariate analysis		Multivariable analysis	
				OR(95%CI)	*p*-value	AOR(95%CI)	*p*-value
Age group (years)					0.078		
≤35	115 (36.9)	77 (67.0)	38 (33.0)	1		–	
> 35	197 (63.1)	112 (56.9)	85 (43.1)	1.5 (0.9–2.5)		–	
Marital status					0.204		
Single/divorced/widow	78 (25.0)	52 (66.7)	26 (33.3)	1		–	
Married/cohabiting	234 (75.0)	137 (58.5)	97 (41.5)	1.4 (0.8–2.4)		–	
Education					0.000		0.002
Illiterate/ primary school	201 (64.4)	106 (52.7)	95 (47.3)	2.6 (1.6–4.4)		2.4 (1.4–7.2)	
Secondary school and higher	111 (35.6)	83 (74.8)	28 (25.2)	1		1	
Household registration					0.036		
Local	92 (29.5)	64 (69.6)	28 (30.4)	0.6 (0.3–0.9)		–	
Non-local	220 (70.5)	125 (56.8)	95 (43.2)	1		–	
Duration of commercial sex work (years)					0.148		
≤2	173 (55.4)	111 (64.2)	62 (35.8)	1		–	
> 2	139 (44.6)	78 (56.1)	61 (43.9)	1.4 (0.9–2.2)		–	
Price charged (RMB)					0.000		0.001
≤30	89 (28.5)	37 (41.6)	52 (58.4)	4.4 (2.4–8.1)		3.8 (1.9–7.6)	
31–50	171 (35.6)	67 (60.4)	44 (39.6)	2.0 (1.2–3.7)		1.8 (1.0–3.3)	
> 50	112 (35.9)	85 (75.9)	27 (24.1)	1		1	
Venues					0.012		
Small beauty salons/massage rooms/guest houses	194 (62.2)	128 (66.0)	66 (34.0)	1		–	
Self-rented room/“market day” building	118 (37.8)	61 (51.7)	57 (48.3)	1.8 (1.1–2.9)		–	
City					0.000		
Liuzhou	119 (38.1)	95 (79.8)	24 (20.2)	1		–	
Pingnan	100 (32.1)	40 (40.0)	60 (60.0)	5.9 (3.3–10.8)		–	
Zhangjiajie	93 (29.8)	54 (58.1)	39 (41.9)	2.9 (1.6–5.3)		–	
Male condom use with new clients in the past month^a^					0.859		
Every time	224 (71.8)	135 (60.3)	89 (39.7)	1.0 (0.6–1.7)		–	
Not every time	88 (28.2)	54 (61.4)	34 (38.6)	1		–	
Male condom use with regular clients in the past month^a^					0.065		0.002
Every time	187 (64.0)	104 (55.6)	83 (44.4)	1.6 (1.0–2.6)		2.4 (1.4–4.2)	
Not every time	105 (36.0)	70 (66.7)	35 (33.3)	1		1	

^a^ The data were drawn from one-month follow-up questionnaire

The average age of participants was 38 years old (range: 18 to 55 years), with 63.1% of participants categorized as older FSWs; Participants who were married or cohabiting accounted for 75.0% of the sample, 64.4% of participants were illiterate or received less than primary school education, and 70.5% of participants had non-local household registration.

In terms of work-related characteristics, more than a half of participants had worked as a FSW for more than 2 years, 64.1% of participants charged less than or equals to 50 RMB per client; and 37.8% of participants solicited clients in self-rented rooms or “market day” buildings, with the remainder of women soliciting clients in small salons, massage parlors or guest houses.

In terms of FC awareness, 17.9% (56/312) of all participants had previously heard of female condom, but only 2 FSW had ever used a FC.

### Patterns of FC use and associated factors

All 312 participants who enrolled in our study completed the one-month follow-up encounter. Among them, 273 (87.5%) participants reported using at least one FC in the past month, and 123 (39.4%) participants had used more than 2 FCs. A total of 920 out of 3120 FCs (29.5%) were used (range: 1–10 per FSW, median: 2). Characteristics of FSW stratified by frequency of FC use (≤ 2 versus > 2 FCs used) are also illustrated in Table 1 (only 39 participants did not use FCs, therefore we combined this group with participants who used less or equal to 2 FCs).

The results of the univariate logistic regression analysis found seven factors that were significantly associated with using more than 2 FCs: age, education, household registration, price charged, venue, enrolled city, and consistent male condom use with regular clients in the past month (Table 1). In the multivariable logistic regression analysis, we found that participants who were less educated (OR: 2.4, 95% CI: 1.4–7.2), charged ≤30 RMB per client (≤ 30 versus 51–80, OR: 3.8, 95% CI: 1.9–7.6; 31–50 versus 51–80, OR:1.8, 95% CI: 1.0–3.3) consistent condom use with regular clients in the past month (OR:2.4, 95%CI:1.4–4.2) were more likely to use FCs (Table 1).

We subsequently stratified the study population by the significantly associated factors identified in our multivariable regression model (education level and price charged per client), and explored patterns of FC use with different types of sexual partners (Table 2). Overall, more participants reported using FCs with regular clients compared with new clients *(p < 0.001)*.

**Table 2:** Female Condom Use with Different Sexual Partners among Participants of Different Characteristics in Three Chinese cities, 2013, *N* = 312

Variables	Total	Price				Education		
	N (%)	≤30	31–50	> 50	*P* value	Illiterate/ primary school	Secondary school and higher	*P* value
FC use with new clients					0.252			0.006
Yes	119 (38.1)	33 (37.1)	48 (43.2)	38 (33.9)		88 (43.8)	31 (27.9)	
No	193 (61.9)	56 (62.9)	63 (56.8)	74 (66.1)		113 (56.2)	80 (72.1)	
Number of used FCs with new clients					0.031			0.003
≤2	279 (89.4)	76 (85.4)	96 (86.5)	107 (95.5)		172 (85.6)	107 (96.4)	
> 2	33 (10.6)	13 (14.6)	15 (13.5)	5 (4.5)		29 (14.4)	4 (3.6)	
FC use with regular clients					0.000			0.000
Yes	220 (75.3)	77 (88.5)	80 (78.4)	63 (61.2)		158 (83.2)	62 (60.8)	
No	72 (24.7)	10 (11.5)	22 (21.6)	40 (38.8)		32 (16.8)	40 (39.2)	
Number of FCs used with regular clients					0.000			0.000
≤2	223 (76.4)	47 (54.0)	79 (77.5)	97 (94.2)		132 (69.5)	91 (89.2)	
> 2	69 (23.6)	40 (46.0)	23 (22.5)	6 (5.8)		56 (30.5)	11 (10.8)	
Future willingness to use free FCs					0.004			0.462
Yes	116 (42.5)	44 (49.4)	42 (37.8)	30 (34.5)		81 (44.0)	35 (39.3)	
No	157 (57.5)	45 (50.6)	69 (62.2)	57 (65.5)		103 (56.0)	54 (60.7)	

When we evaluated the subgroup of FSW charging ≤30RMB per client, we found that more participants (88.5%) had used FCs with regular clients compared with their counterparts who charge 31-50RMB (78.4%) and 51-80RMB per client (61.2%) *(p < 0.001)*. There were no differences in FC use between the three groups with new clients.

Evaluation of participants based upon level of education showed that a higher proportion of participants with lower levels of education used FCs with new clients (43.8%, *p = 0.006)* and regular clients (83.2%*, p < 0.001)* compared to those with higher levels of education.

### Discomforts associated with FC use

Among the 273 participants who reported using FCs during the course of the study, 71.0% participants reported discomfort from FCs use **(**Table 3**)**. The most frequent discomfort reported was: “Discomforts with the outer ring” (76.4%, 149/195), “[FCs] feel itchy and painful inside the vagina” (33.8%, 66/195), and “The FC is too noisy during intercourse” (23.6%, 46/195).

**Table 3:** Discomforts Associated with FCs use, *N* = 195

Variables	n (%)
Suffered from pain from the outer ring	59 (32.8)
Had difficulties with penis insertion	19 (9.7)
The FC slipped out of the vagina during intercourse	7 (3.6)
The FC feels itchy and painful inside the vagina	66 (33.8)
The FC is too noisy during intercourse	46 (23.6)
Difficulty removing the FC after intercourse	14 (7.2)
Encountered breakage of FC	9 (4.6)

### Future willingness to use FCs

Among the 273 participants who reported using FCs during the course of the study, 116 (42.5%) expressed willingness to use the device again in the future (Table 2). Participants who charged ≤30RMB exhibited the highest proportion (49.4%) compared with the other two groups (31–50 RMB: 37.8%; 51–80 RMB: 35.5%) *(p = 0.004)*. There was no difference in willingness observed among participants with different levels of education.

Of the participants who had used 1–2 FCs during the month, 22.3% were willing to use it again in the future, compared with 65.9% of those who had used more than two FCs during the same time period *(p < 0.001)*.

The reasons participants provided for unwillingness to use FCs in the future included: “[The FC] is complicated and not convenient to use” (75.8%, 119/157), “I prefer to use male condoms” (49.0%, 77/157), “[FCs] feel uncomfortable inside the vagina” (34.4%, 54/157), “My clients refused to use [FCs]” (31.8%, 50/157).

Among the 39 participants who did not use any FCs during the course of the study, all reported that they will not use FCs in the future (Table 2). The reasons are reported as follows: “[The FC] is complicated and not convenient to use” (61.5%, 24/39), “I prefer to use male condoms” (61.5%, 24/39), “My clients refused to use [FCs]” (38.5%, 15/34).

## Discussion

Our study provides important insight into the acceptability of FCs among low-fee FSWs, a subgroup that has not been previously highlighted in FC intervention studies in China. Overall, 87.5% of participants used at least one of the FCs that were distributed during the study period, and 42.5% of participants who used the distributed FCs expressed willingness to use them again in the future. This proportion is greater than or equal to percentages reported in previous studies conducted in China [5, 14–18]. Furthermore, we observed differences in patterns of FC use based upon participants’ level of education and price charged per client, which also provides an indication of which sub-populations should be targeted for future FC promotion.

We found that participants who charged less per client were more likely to use FCs. The lowest-fee FSWs (≤30RMB per client) have been shown previously to be older compared with FSW working in other venues, and to have an older client base as well [7]. Shaobing Su et al., performed a study with 1022 low-fee FSW in southwestern China, and found that women in this group experience difficulties negotiating male condom use due to a higher prevalence of erectile dysfunction among clients combined with a lower level of awareness regarding HIV risk [20]. By contrast, FCs do not require an erect penis to function, which may ease the anxiety of older clients and therefore FSWs with this subset of clients with an alternative method to protect themselves [22].

We also found that low-fee FSW in our study who were less educated were more likely to use FCs. This finding may reflect that low-fee FSWs with higher levels of education feel more empowered to negotiate male condom usage as has been demonstrated in previous studies from Zimbabwe and Armenia [23, 24], and therefore may perceive less need to adopt the more complex FC when they are already successfully negotiating and using male condoms [5, 25].

Based upon these findings, low-fee FSWs who are less educated and charge the lowest fees per client may be appropriate initial candidates for FC promotion in future HIV prevention efforts in China. A number of factors ought to be considered if FCs are included as an additional barrier method in Chinese HIV prevention programs in the future. First, it may be most practical for interventions to initially focus on strategies for enabling low-fee FSWs to negotiate FC use with regular clients who refuse to use male condoms: we found that regardless of educational background and price charged, the participants were more likely to both try FCs and use FCs more frequently with regular clients. This is consistent with a study from Central America showing that the highest proportion of FC use occurred with regular clients [26]; Additionally, in our study, participants who reported using male condoms with regular clients every time were also more likely to use FCs. This indicates that they may feel more at ease communicating about and negotiating condom use when regular clients refuse or are unable to use male condoms. Finally, some studies have found that to increase the likelihood of success during FC negotiation, FSW can be taught verbal strategies, such as, “The female condom will feel less constricting and more comfortable than a male condom” or, “Sex using a female condom may feel like a condom is not being used” [13, 26].

Second, proper technical training in FC use is paramount, as evidenced by the high number of reports in the literature, and in our cohort, regarding technical discomforts related to FCs that could have been corrected or avoided with additional instruction [27, 28]. To introduce FC use more visually, a female pelvic model can be used to allow participants to practice inserting and removing the FC themselves. This will also provide FSW with an opportunity to discuss difficulties using FCs with outreach workers [29]. In addition, proper usage is often not immediately or intuitively understood after just one instruction. A total of 71.0% participants who reported using FCs in our study reported discomfort from FCs use. This may indicate improper usage and suggest a need for more frequent training. Indeed, the failure of the “first try” can subsequently discourage users from trying the FC again. In our study, 88.9% of participants who used only one female condom showed unwillingness to use FCs again in the future. Instead, a follow-up visit with an outreach worker after the “first try” may help overcome this hurdle. Randomized trials conducted by Beksinska et al. showed that, there was an overall reduction in all failure modes, aside from clinical breakage, from the first to the subsequent FC use, regarding four FCs (FC2, Woman’s Condom, Cupid and VA w.o.w) [30].

Finally, in this study, we offered FCs free of charge, and explored the acceptability of FC use among low-fee FSW based upon a free supply assumption. The over-the counter price of a FC is 15 RMB each, making FCs too expensive for low-fee FSWs to purchase. If participants had been asked to pay for the FCs, we anticipate that few low-fee FSWs would have actually used FCs. Therefore, FC promotion efforts should be publicly subsidized and targeted specifically for FSW for whom male condoms may be less feasible or practical to use (e.g. low-fee FSW). These efforts would ideally be integrated into larger-scale efforts to promote male condom usage so as to maximize efficiency and cost-effectiveness of outreach efforts and resources. Global public policymakers can also advocate for more manufacturers to produce FCs, negotiate better prices, and develop effective promotion and distribution programs [31].

### Limitations

Our study is limited by the short duration of follow up, which may impact our ability to assess the long-term acceptability of FC use among this population. However, we believe that if anything, this would have underestimated the magnitude of our findings. Over time, if one considers a framework such as Diffusion of Innovations, it is possible that as FSWs begin to hear positive feedback regarding FCs from co-workers who have successfully used FCs (i.e. the early-adopters), participants who were initially uninterested in using FCs may be motivated to try FCs. Future longer-term studies would benefit from utilizing theoretical frameworks such as Diffusion of Innovations to provide additional insight into the dynamics of FC adoption of across populations of FSW, which can also guide strategies for HIV/STI prevention programs. The second limitation of the study was its reliance on manually counting used FC packages for measurement, without parallel validation techniques such as interviewing clients, or requiring a self-reported coital log. It is possible that some participants forgot to put their used packages into the plastic bag, underestimating the number of FCs they have used.

## Conclusions

We found that low-fee FSWs who are less educated and charge the lowest fees per client may be appropriate initial candidates for FC promotion in future HIV prevention efforts in China. Strategies to consider include teaching FSWs tactics for negotiation of FC use that can be applied first with regular clients and later with new clients, and providing education and support through local outreach workers to maximize ease-of use, minimize discomforts and troubleshoot difficulties that arise with FC usage.

## Additional file

Additional file 1: Interview Questionnaire (Baseline and follow-up visit). (DOCX 33 kb)

## Data Availability

The datasets used and/or analyzed during the current study are available from the corresponding author on reasonable request.

## Abbreviations

CDC: Center for Disease Control and Prevention
CI: Confidence interval
FC: Female Condoms
FSWs: Female Sex Workers
