# Supplementary material for: Short-term acceptability of female condom use among low-fee female sex workers in China: a follow-up study
Source: BMC Womens Health. 2019 Jun 14;19:77. doi: 10.1186/s12905-019-0773-7 (PMC6570840; doi:10.1186/s12905-019-0773-7)
Supplement: Supplementary file 1 — Interview Questionnaire (Baseline and follow-up visit). (DOCX 33 kb) [file 12905_2019_773_MOESM1_ESM.docx]

| **Interview Questionnaire (Baseline Survey)** | | |
| --- | --- | --- |
| Name (Optional)**:** | | Cell Phone Number： |
| Name of Work Venues： | | Survey Date: ____Year______Month______Day |
| Type of Work Venues: | | 1. Small beauty salons/massage rooms/guest houses 2. Self-rented room/”market day” building |
| City: | | ID: |
| **QID** | **QUESTION** | **CHOICES** |
| **A** | **Basic information** | |
| A01 | Date of birth？ | ________Year |
| A02 | Marriage？ | 1. Single 2. Married 3. Cohabiting 4. divorced/widow |
| A03 | Education？ | 1. Illiterate 2. primary school 3. Secondary school 4. High school and higher |
| A04 | Household registration? | 1. Local 2. Non-local |
| A05 | Duration of commercial sex work (years)? | 1. ＜3 months 2. 3-6 months 3. 6-12 months 4. 12-24 months 5. >24 months |
| A06 | Price charged？ | _______RMB |
| A07 | Have you ever heard of Female Condoms? | 1. Yes 2. No (questionnaire finished) |
| A08 | Have you every used Female Condoms? | 1. Yes 2. No |

| **Interview Questionnaire (One-month Follow-up Survey)** | | | |
| --- | --- | --- | --- |
| Name (Optional)**:** | | | Cell Phone Number： |
| Name of Work Venues： | | | Survey Date: ____Year______Month______Day |
| Type of Work Venues: | | | 1. Small beauty salons/massage rooms/guest houses 2. Self-rented room/”market day” building |
| City: | | | ID: |
| **QID** | **QUESTION** | | **CHOICES** |
| **B** | **Use of female condoms in the past month** | | |
| B01 | In the past month, have you ever used male condom with new clients? | 1. Yes, every time.  2. Yes, but not every time | |
| B02 | In the past month, how many female condoms have you ever used with new clients? | 1. __FCs 2. Have not used FCs at all. | |
| B03 | In the past month, have you ever used male condom with regular clients? | 1. Yes, every time.  2. Yes, but not every time  3. I have no regular clients. | |
| B04 | In the past month, how many female condoms have you ever used with regular clients? | 1. __FCs 2. Have not used FCs at all. | |
| **C Difficulties to use FCs** | | | |
| C01 | Suffered from pain from the outer ring | 1. Yes 2. No | |
| C02 | Had difficulties with penis insertion | 1. Yes 2. No | |
| C03 | The FC slipped out of the vagina during intercourse | 1. Yes 2. No | |
| C04 | Encountered breakage of FC | 1. Yes 2. No | |
| C05 | The FC feels itchy and painful inside the vagina | 1. Yes 2. No | |
| C06 | The FC is too noisy during intercourse | 1. Yes 2. No | |
| C07 | Difficulty removing the FC after intercourse | 1. Yes 2. No | |
| **D Future willingness** | | | |
| D01 | Would you like to use Female Condoms in future? | 1. Yes (questionnaire finished) 2. No | |
| C02 | Why don’t you want to use Female Condoms in future? | 1. I prefer to use male condoms 2. The[FC] is complicated and not convenient to use 3. [FCs] feel uncomfortable inside the vagina 4. My clients refused to use 5. Other reasons, please explain _________ | |
